# Targeted genome sequencing for tuberculosis drug susceptibility testing in South Africa: a proposed diagnostic pipeline

**DOI:** 10.1099/acmi.0.000740.v3

**Published:** 2024-02-16

**Authors:** Tayarv J. Bagratee, David J. Studholme

**Affiliations:** ^1^​ Biosciences, University of Exeter, Exeter, UK; ^2^​ Department of Anatomical Pathology, Inkosi Albert Luthuli Central Hospital, Durban, KwaZulu-Natal, South Africa

**Keywords:** tuberculosis, tNGS, antimicrobial resistance, genomics, sequencing

## Abstract

In July 2023, the World Health Organization (WHO) began recommending targeted next-generation sequencing (tNGS), due to its ability to detect resistance to many drugs with a single test. In March 2023, South Africa further adopted the GeneXpert XDR cartridge, which detects mutations associated with resistance to second-line injectable drugs. Here, we consider the feasibility for implementing tNGS in South Africa, what such a facility might look like and the specific context of this upper-middle-income country. Whilst the WHO now recommends tNGS for TB diagnostics and DST, there are many economic and infrastructural challenges opposing its deployment. In lieu of this, we instead recommend a stratified diagnostic pipeline that utilizes South Africa’s existing GeneXpert technologies, attempting to reduce the costs associated with implementation of tNGS.

## Data Summary

No new data were generated in this study.

## Introduction

Tuberculosis (TB) continues to pose a major global health threat that is worsened by rising antimicrobial resistance [1]. Reduced efficacy of antibiotics leads to increased morbidity, mortality, prolonged hospitalization, and the adverse effects of being exposed to second-line anti-TB drug regimens [2, 3]. Accurate diagnosis is challenging. For example, immunocompromised patients with decreased sputum bacillary load may yield false negative results. Patients with latent or reactivation TB may yield false positives [4, 5]. South Africa is a hot zone for multidrug-resistant (MDR) and extensively drug-resistant (XDR) TB and in 2019 had the highest incidence of co-infection of TB with HIV worldwide [6].

Drug susceptibility testing is essential for the effective management of tuberculosis and genotype-based prediction offers a promising alternative to phenotypically testing the susceptibility of cultured bacteria. The WHO Catalogue of Mutations [7] provides a list of genetic variants within the *Mycobacterium tuberculosis (MTB)* genome that are predictive of drug resistances. Genotyping may be either untargeted via genome sequencing or targeted via amplification of specific genomic loci.

In July 2023, the World Health Organization (WHO) began recommending targeted next-generation sequencing (tNGS), due to the ability of a single test to detect resistance to multiple drugs. Furthermore, tNGS was faster than culture-based approaches for drug sensitivity testing (3 to 5 days versus 4 to 6 weeks). However, the WHO acknowledges that the advantages of tNGS are highly context-dependent, and utilization of tNGS may be feasible only in centralized settings, due to the challenges involved with setup, consumables and infrastructure [8]. Here, we consider the feasibility for implementing tNGS in South Africa and what such a facility might look like in the specific context of this upper-middle-income country.

## Current diagnostic methods for TB in South Africa

The current mainstay for TB diagnosis in South Africa is the Cepheid GeneXpert Ultra system, which uses polymerase chain reaction (PCR) and molecular beacon analysis of the *rpoB* gene. It offers a rapid turnaround time and detects resistance to the first-line antimicrobial drug rifampicin, with a high sensitivity [9]. In March 2023, South Africa further adopted the GeneXpert XDR cartridge, which detects mutations predictive of resistance to second-line, injectable drugs. If the GeneXpert Ultra returns a positive result for rifampicin resistance, this sample will automatically have a GeneXpert XDR performed. The current diagnostic pipeline takes 1 day to confirm the diagnosis of TB and/or rifampicin resistance. Two days are required for a diagnosis of XDR TB. A total of 6 weeks is required to receive a result from culture, confirming presence of TB; an additional week is required for drug-susceptibility testing.

Other diagnostic methods are also used, depending on the suspected organ system infected by TB. Different protocols are used within different institutions around South Africa. Almost all patients receive a chest X-ray, GeneXpert assay and bacterial culture with drug-sensitivity testing. To hasten the administration of treatment when pulmonary TB is suspected, GeneXpert and culture are performed simultaneously on the same sputum sample. This provides a rapid GeneXpert result (within days), whilst awaiting the much slower culture results (weeks). Disseminated TB (or extrapulmonary TB) presents with completely different symptoms and the patient’s sputum may not harbour TB bacilli. In these patients, diagnosis typically involves a biopsy/tap of the infected site, GeneXpert, microscopy and culture, all on the same sample.

## Potential advantages of sequencing-based prediction of drug susceptibility

The main advantage of genotype-based prediction over culture-based testing is the rapid return of results. For example, it can take up to 15 weeks to complete susceptibility testing for first- and second-line drugs in a reference laboratory in the United Kingdom [10], including up to 3 weeks of testing in a Mycobacterial Growth Indicator Tube (MGIT) and a further four to eleven weeks of culture testing if there is resistance to first-line drugs. In South Africa, a minimum of 6 weeks is needed for susceptibility testing in culture, extending by a further 6 weeks if resistance to first- and second-line drugs is identified. However, genotyping could provide a complete resistance profile for all TB regimen drugs after about 2 weeks, by combining MGIT culture and whole-genome sequencing [10]. Targeted genotyping of samples extracted from sputum would bypass the need for culturing in MGIT, thus saving more time.

Aside from accelerating delivery of results, genotype-based prediction can be more accurate than culture-based testing, in some cases. The mutation S450L in *rpoB* confers drug resistance but also slows growth in culture and was the most common variant confirming rifampicin-resistance in a study performed in Khayelitsha, South Africa [11]. Since growth rate is a surrogate marker for resistance, the S450L mutation this may result in a false-positive result for susceptibility in culture while sequencing will correctly predict resistance to rifampicin [10, 12, 13].

On the other hand, genotype-based prediction of resistance can have levels of sensitivity as low 72.3 % for pyrazinamide, 70.4 % for ethionamide and 62.9 % for moxifloxacin [7]. The latter two are used in the treatment of MDR (multi-drug resistant) TB. Therefore, incorrectly inferring resistance to ethionamide and moxifloxacin will lead to inappropriate upscaling of TB treatment, thus exposing the patient to more dangerous XDR-regimen drugs with a greater incidence of adverse side effects, and poorer efficacy [14, 15].

## Practical considerations for targeted sequencing (tNGS) from sputum samples

The lengthy procedure of culturing can be avoided by targeted PCR amplification of specific genomic loci directly from sputum samples and sequencing the amplicons by next-generation sequencing (NGS) [16]. This strategy, known as targeted NGS (tNGS), is recommended by WHO as a rapid means of combined TB diagnosis and drug sensitivity testing (DST). In addition to the time saving, tNGS incurs lower sequencing costs than whole-genome sequencing and the ability to detect rare variants. However, implementing tNGS on sputum samples presents some challenges; the sample needs to be inactivated and bacterial DNA must be extracted.

### Inactivation of pathogenic bacteria in samples

Samples from suspected TB patients must be inactivated by ethanol or heat for safe handling in the laboratory [17]. Heat-killing is not always adequate, and a combination of ethanol and heat-inactivation has been proposed [18]. Chloroform combined with 70 % ethanol prior to heat-inactivation at 80°C led to no regrowth of TB bacilli and minimal degradation of the DNA material, such that quality was adequate for sequencing on the Illumina platform [18].

### DNA extraction, purification and amplification

Methods for DNA extraction from sputum prior to tNGS include magnetic bead chemistry, thermolysis, chemical-based methods and enzymatic-based methods [19]. Before implementing tNGS, it is important to consider the requirements for these steps in the workflow. Sputum samples acquired from patients are admixed with DNA from patient and from the microbiota of the respiratory tract [20]. Decontamination of the sample involves purification to remove non-TB DNA. Amplification of the residual TB DNA can also be performed alongside this to improve the diagnostic yield [21].

One example utilises the Maxwell purification kit and machine to purify their sputum sample, and the Agencourt AMPure XP magnetic beads to purify the amplicons in their assay [22]. In another setting, researchers performed DNA extraction using heat shock extraction, and the GeneLEAD VIII system, which utilises DNA adsorption to magnetic particles following cell lysis [23].

Choosing a method of extraction would ultimately depend on the cost of setup and the amount of TB DNA extracted. One study [24] developed a scoring system to analyse kit-based approaches for MTB DNA extraction; the highest-scoring product was the Genolution Nextractor, which has a per-specimen cost of $2, and an instrument cost of $12 000. This was lower in comparison to other instruments assessed (a total of 17 instruments were assessed, ranging from $3000 to over $100 000). Whilst this may be an attractive option for deployment in South Africa, those authors identified a limitation in their study being that DNA extraction efficiency was not measured [24]. Amplification is an optional step that can be performed if the yield from DNA extraction is minimal, with some library prep kits performing amplification automatically [25, 26].

### WHO-approved tNGS assays

The WHO 2018b technical guide [27] outlines two proposed methods for tNGS directly from sputum, namely the Next Gen-RDST and Deeplex-MycTB assays. The WHO further recommends the NanoTB from Oxford Nanopore Technologies and the TBseq from ShengTin Biotech. However, there is not yet a body of published research using these products. Minimal literature is published at present regarding the NanoTB product, and the TBseq is only WHO-approved for resistance to a single drug (ethambutol) [8]. Thus, the only assays worth evaluating at present are the Deeplex Myc-TB assay and Next-Gen RDST.

### How do the Deeplex Myc-TB and Next Gen RDST assays perform when compared to the current diagnostic pipeline?

The Next-Gen RDST tests fewer genetic locations than the GeneXpert XDR, whilst also being $10 more expensive per sample. This makes it an objectively poorer option in comparison to the current diagnostic timeline. The Deeplex Myc-TB assay tests for nine more drugs than the GeneXpert XDR. However, it costs $118 more (excluding the costs to set up NGS). The cost for TB culture and DST is approximately $13 [28, 29]. Providing tNGS for every sample at this price point would be immensely expensive. This supports the notion of a stratified diagnostic pipeline that utilizes the existing TB-diagnostic infrastructure to stratify cases appropriately (see Table 1).

**Table 1.** Drugs tested for by GeneXpert Ultra, GeneXpert XDR, NextGen RDST and Deeplex Myc-TB

Drug		GeneXpert Ultra	GeneXpert XDR	NextGen RDST	Deeplex Myc-TB
**First-Line**	Rifampicin	✔	✘	✔	✔
	Isoniazid	✘	✔	✔	✔
	Pyrazinamide	✘	✘	✘	✔
	Ethambutol	✘	✘	✘	✔
	Streptomycin	✘	✘	✘	✔
**Second-Line**	Amikacin	✘	✔	✔	✔
	Ethionamide	✘	✔	✘	✔
	Linezolid	✘	✘	✘	✔
	Kanamycin	✘	✔	✔	✔
	Capreomycin	✘	✔	✔	✔
	Ofloxacin	✘	✔	✔	✔
	Moxifloxacin	✘	✔	✔	✔

In the existing pipeline, the GeneXpert XDR cartridge allows a clinician to escalate a patient’s drug regimen from MDR, to XDR and extremely drug resistant (XXDR) TB regimens. In the time that a patient is empirically covered, a clinician can await the results for traditional culture-based DST and thereafter de-escalate and narrow therapies appropriately when these results are obtained. However, the time to a DST and culture result is minimum of 7 weeks, whilst a Deeplex Myc-TB result could take potentially only 3 weeks [10].

Based on the above findings, the Deeplex Myc-TB assay appears to be the most promising option for tNGS. Whilst the WHO believe that tNGS may be more cost effective if it results in earlier effective treatment [8], they do not provide evidence of an improvement in patient outcomes with use of tNGS (e.g. time to treatment, or treatment outcomes) [8]. The primary proposed benefit afforded by tNGS is the earlier progression to targeted antimicrobial therapy, if multi-drug resistance is detected. From a public health and economic standpoint, cost saving is awarded by potentially mitigating hospitalization due to the progression of disease from MDR to XDR and XXDR TB. Cost savings are further improved by reducing the need to treat adverse side effects of anti-XDR-TB drug regimens, whilst also potentially reducing the spread of XDR TB.

## How might tNGS be incorporated into the diagnostic pipeline of TB?

Cost of tNGS increases with number of samples processed. A pragmatic strategy might be performing tNGS only for samples that return a rifampicin-resistant result from the GeneXpert Ultra. There is likely to be a good response to first-line treatment for patients whose GeneXpert test results is drug-susceptible (i.e. not rifampicin-resistant or XDR) TB. During the wait for a GeneXpert XDR result, tNGS should already be underway as usage of the Deeplex Myc-TB assay will provide DST for nine additional drugs. Thus, all the benefits that come with the speed of a DST result provided by tNGS are achieved, with only a 1 day delay in processing a GeneXpert Ultra result.

**Fig. 1.**
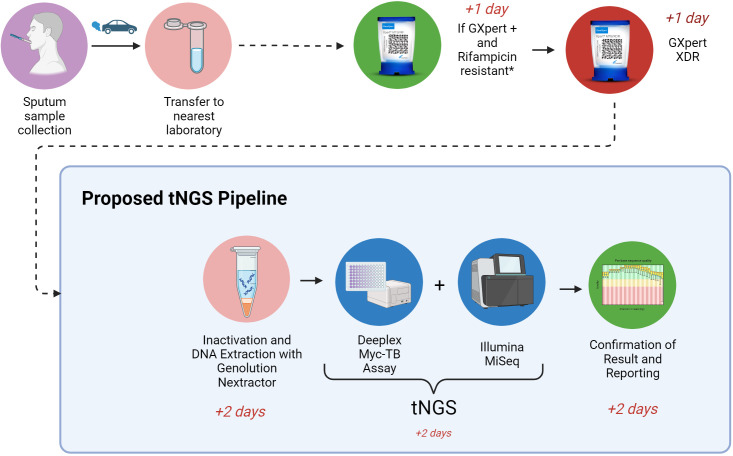
Proposed tNGS pipeline. Image created with BioRender.com.

This approach would result in a significant cost reduction for the implementation of tNGS, as the number of samples processed will be reduced, and a cheaper sequencing machine could be used due to the lower throughput (e.g. Illumina iSeq). The most cost-effective approach to tNGS would include: 1) stratification of TB samples, submitting only MDR +cases for tNGS. 2) Ethanol +heat inactivation. 3) DNA extraction with the Genolution Nextractor. Finally, 4) tNGS by use of the Deeplex Myc-TB and Illumina MiSeq (Fig. 1). Whilst tNGS samples may be stratified by this method, it is important to note that all patients should still receive a culture-based DST (regardless of the GeneXpert result), so as not to miss drug-resistances not detected by the GeneXpert.

## Challenges to the deployment of tNGS in South Africa

The major challenges to deploying a tNGS pipeline include cost and infrastructure. The main setup costs include purchase of instruments for assays and sequencing. Additional costs include consumables, infrastructure, and staff training (Table 2). Recent implementation of whole-genome sequencing for TB in a low-middle income country with a high burden of MDR-TB encountered costs including shipping and customs, annual maintenance, and sequencing library preparation kits [30].

**Table 2.** Costs associated with the setup of tNGS in South Africa

**Workforce**	Training of staff Continued employment of staff and salary packages
**Infrastructure**	Electricity and backup power Maintenance and service Internet and phoneline services Cloud services and storage Floor/building/office space
**Sequencing**	Initial purchase of Illumina MiSeq or iSeq Service-level agreement for sequencing platform Deeplex Myc-Tb Kits Miscellaneous consumables
**Location**	Ideal rollout would cover nine labs (one for each province).

South Africa faces challenges in resources and infrastructure. The recent SARS-CoV-2 pandemic placed tremendous financial burden on this country [31] whose economy has been gradually weakening year after year [32–34]. Metropolitan areas are separated by vast areas of rural villages and townships. Citizens residing in wealthier areas have easier access to privatised healthcare and treatment, and a reduced TB burden [35]. High TB infectivity is directly linked to poverty and overcrowding [36]. Areas with a higher TB burden are of a lower socio-economic status (SES) and have limited access to healthcare. Deploying tNGS in these areas would not be feasible due to the poor infrastructure. Furthermore, TB is widespread throughout the entire country.

The World Health Organization appears to concur with these sentiments, acknowledging that tNGS involves a highly complex workflow, and that challenges will be encountered in multiple areas of setup including: internet connectivity, data storage, data management and installation [8]. South Africa further suffers from massive, prolonged power outages (load-shedding), both scheduled and unscheduled. Whilst many laboratories have generators and alternate means of power, these episodes of load-shedding still result in sudden losses of power prior to the rerouting of electricity from an alternate source. These sudden losses of power will reduce the operational times of the equipment and could further result in critical system failures and loss of data.

Laboratory diagnostics in the South African public sector are rendered by the National Health Laboratory Services (NHLS). If tNGS were to be implemented, the ideal sites would be the reference laboratories attached to tertiary institutions, due to the existing support and infrastructure.

### Shortcomings in the current diagnostic pipeline

There is no evidence yet that GeneXpert has significantly impacted TB-related morbidity, mortality, and time to treat. Potentially beneficial amendments to the TB diagnostic pathway have been proposed, given the shortcomings of the existing pipelines and suboptimal GeneXpert implementation. Other platforms for tNGS may be similarly ineffective, as the failure is attributable to frequent lack of patient follow-up rather than diagnostic modality. Thus, deployment of any tNGS would likely require the same additional complementary investments as those recommended for GeneXpert [29].

### Comparison of potential tNGS rollout in South Africa, to other low- and middle-income countries

The difficulties faced in implementing tNGS are ubiquitous amongst all low- and middle-income countries. A recent strategy that has been successfully implemented in Nigeria utilised a model comprising preparation, implementation, and sustainability phases [37, 38]. The preparation phase involved site and staff identification, establishing a timeline with achievable goals, with effective co-ordination, communication and contracting. The implementation and sustainability phases involved setting up sequencing and laboratory protocols, purchasing of supplies and staff training. They also suggest stratification of TB samples, and usage only of samples that have already been confirmed as drug resistant. Given the similar TB burden between South Africa and Nigeria, this model may prove useful in the rollout of tNGS in South Africa. The timeline suggests that setup of a single institution may take 2 years to complete, however due to delays due to the COVID-19 pandemic their rollout was extended to 4 years.

A recent feasibility study in an Indian public health reference laboratory concluded that tNGS was feasible in reference laboratories, but acknowledged that work-streams require further optimisation [39]. The setting of their study invites extrapolation of their conclusions to South Africa, given the marked similarities to the TB burden, the presence of TB hotspots, and the diagnostic pipeline in India [40], but with the caveat that incidence of tuberculosis is significantly lower in India than in South Africa, according to WHO statistics. The study included samples already tested positive with GeneXpert, and the chosen tNGS technology was the Deeplex-MycTB kit. The authors document many challenges, including batching of samples, leading to delays in turnaround times. The Indian feasibility study noted a cost of $230 per sample and further observed a lack of data on the per-sample cost of tNGS, as many studies do not include all steps (library prep, DNA extraction, purchase of reagents) in the cost calculation. That study did not factor-in an initial cost of purchase of a sequencing instrument, as an Illumina MiSeq had been donated to one of their reference laboratories.

A national survey [22] performed in DR Congo between 2016 and 2017, performed NGS of 1708 samples, without use of culture. The survey found reliable results, with poor outcomes only seen in cases with a low bacillary load or sample transport problems. It further demonstrates that NGS is possible in a lower-income country, and recommends it due to the benefits offered logistically as rapid sputum transport with maintenance of a cold chain is not required.

### Public health benefits associated with tNGS rollout

The benefits associated with tNGS extend beyond TB diagnostics [37, 38]. The implementation of NGS and tNGS infrastructure will upskill the molecular workforce of the country. NGS instruments may also be utilised in the research and diagnostics of other bacterial and viral genomes, improving public health in South Africa. The Deeplex Myc-TB assay also provides SNP-based lineage identification, allowing for the tracking of disease spread and hotspot identification.

Molldrem *et al*. performed a qualitative study interviewing patients, doctors, and policy stakeholders [41]. Their study found universal support in the progression to NGS, recommending that lessons learnt from previous NGS initiatives should be used in the rollout of nationwide NGS. Participants were identified to be supportive of sharing patients’ deidentified data for public health surveillance and research purposes. They further recommend rigorous community engagement, capacity-building and education regarding the development of NGS facilities.

## Conclusion

Whilst the WHO now recommends tNGS for TB diagnostics and DST, there are many economic and infrastructural challenges opposing its deployment. We instead recommend a stratified diagnostic pipeline that utilises South Africa’s existing GeneXpert technologies in an attempt to reduce the costs associated with implementation of tNGS. The benefits associated with GeneXpert systems (rapid diagnosis and DST within 48 h), and culture-based DST (gold-standard for DST), are invaluable. Removing these elements completely from the TB diagnostic pipeline and moving exclusively to tNGS would be ill-advised. Deployment of tNGS in South Africa should be implemented with the recommendations provided by previous pilot studies performed in other low- and middle-income countries. Proceeding the rollout of tNGS, it should further be followed by research into its impact on patient outcomes, assessing whether there are truly reduced times to targeted therapy, and reduced morbidity and mortality associated with XDR and XXDR TB.
